# Supplementary material for: When winning costs your peace: How does vertical individualism Hijack relaxation capacity? Network analysis and mediation models
Source: PLoS One. 2025 Nov 6;20(11):e0335851. doi: 10.1371/journal.pone.0335851 (PMC12591470; doi:10.1371/journal.pone.0335851)
Supplement: Attachment — Submitted filename: 5.22_letter to reviewer.docx [file pone.0335851.s007.docx]

**Dear Editor,**

Thank you for your guidance. I have made the necessary revisions to the manuscript based on your suggestions. Specifically, I have added the exact study timeframe in the Methods section (2025.3.17–2025.4.17), which is now reflected in line 136.

Regarding the second suggestion, I would like to clarify that the questionnaire was distributed via the Naodao researcher platform. On the platform's homepage, I included the Other-informed consent form, which was uploaded to the system. Participants were required to consent before proceeding to the questionnaire, and this process is documented within the system.

Regarding the involvement of minors, I would like to highlight that one of the 550 valid participants was 17 years old (Participant number: 339). We identified this at the time of participation and took the necessary steps to contact their parents and counselors for verbal consent. All other participants were 18 years or older.

Please let me know if you need any further details or clarifications.

Sincerely,

Haoyu Wang
