# Supplementary material for: When winning costs your peace: How does vertical individualism Hijack relaxation capacity? Network analysis and mediation models
Source: PLoS One. 2025 Nov 6;20(11):e0335851. doi: 10.1371/journal.pone.0335851 (PMC12591470; doi:10.1371/journal.pone.0335851)
Supplement: Attachment — Submitted filename: comments to the authors.docx [file pone.0335851.s006.docx]

This study employed samples from the Chinese university student population to investigate the psychological mechanisms linking cultural values to rest intolerance through status anxiety. The research question carries substantial theoretical and practical significance, particularly in China's highly competitive educational environment, making the examination of cultural and psychological factors contributing to rest intolerance particularly timely. The advantage of this study lies in its first exploration of the causes of rest intolerance in the Chinese environment, although only exploratory. However, the manuscript would benefit from addressing the following concerns:

1 In lines 67-71, the author states that "This conflict may significantly intensify the experience of rest intolerance." However, from my perspective, individual achievement itself can be viewed as a means of contributing to the collective. For instance, pursuing personal development goals appears compatible with fulfilling collective obligations, suggesting individualism and collectivism may not necessarily contradict each other.Moreover, while the author emphasizes this conflict's impact on rest intolerance, I question whether this is indeed the primary factor. In my view, the causes are likely multifaceted - including family upbringing, cultural environment, and even individualism itself. The conflict mentioned by the author doesn't appear to be a definitive causal factor.

2 The statement in line 87, "The relationship between cultural values and rest intolerance may be influenced by additional factors," clearly suggests a moderating effect rather than mediation as the author claims. When variable B affects the relationship between A and C, this conceptually represents moderation (a conditional effect), not mediation (an intervening mechanism). The author appears to have misapplied these fundamental statistical concepts in their interpretation.

3 In lines 157-158, it appears the author intended to refer to "horizontal individualism, vertical individualism, horizontal collectivism, and vertical collectivism" (rather than the terms currently presented in the text).

4 Regarding lines 214-215, please maintain consistent terminology for the dependent variable throughout the manuscript. While "rest intolerance" may be alternatively referred to as "rest shame" in Chinese academic contexts, the English version should use a single standardized term (preferably "rest intolerance") for reader comprehension. This inconsistency also appears in lines 217 and 219. We recommend conducting a thorough proofreading of the entire manuscript to ensure terminological consistency for this key construct.

5 Please revise the legend for Figure 3. The current inclusion of "Rest Intolerance" in the figure legend appears functionally unnecessary as it does not contribute meaningful explanatory value to the graphical presentation.

6 In line 217, the author states "a positive correlation between resting shame and status anxiety," implying a positive association between rest intolerance and status anxiety. However, in line 177, the author specifies that "Positive and negative associations are represented by blue and red edges, respectively." This creates a contradiction, as the figure depicts rest intolerance and status anxiety connected by a red edge (indicating a negative correlation), which is inconsistent with the textual description.

7 The phrasing in lines 218-219 – "a negative correlation between resting shame and status anxiety and horizontal collectivism" – is structurally ambiguous and may lead to misinterpretation. It remains unclear whether: 1)resting shame correlates negatively with both status anxiety and horizontal collectivism, or 2) resting shame and status anxiety collectively correlate negatively with horizontal collectivism.

8 The centrality analysis in Section 3.3 attempts to identify the most central node in the network, but unfortunately fails to achieve its intended purpose. Rather than examining global network centrality - which is inherently influenced by node selection bias - the analysis should focus on bridge centrality metrics between the rest intolerance and individualism-collectivism subnetworks, as these would provide more meaningful insights into the core research questions. The authors must further justify their node selection strategy, particularly explaining why rest intolerance was treated as a unitary construct rather than being operationalized through its constituent dimensions. A critical remaining question is how the network structure and centrality findings might differ if the multidimensional components of rest intolerance were modeled as separate nodes in the analysis.

9 Why not control for covariates in network analysis?

10 While the use of cross-sectional mediation in this exploratory study may be marginally acceptable, the proposed mediation pathways among individualism-collectivism, status anxiety, and rest intolerance hold limited substantive value. Given that cross-sectional mediation can produce statistically significant effects based solely on correlations—without establishing temporal precedence or causal direction—the reported findings require stronger theoretical justification.

11 As a non-specialist in cultural value research, I find it unclear whether individualism-collectivism can adequately serve as a representative measure of cultural values, or how precisely these two constructs differ and relate to each other conceptually. This distinction is particularly important as the manuscript appears to use these terms interchangeably without proper clarification, potentially conflating the specific dimension of individualism-collectivism (which focuses on self-group relations) with the broader, multidimensional construct of cultural values that encompasses various societal orientations beyond just this single aspect. The authors should explicitly address this conceptual overlap and justify their operationalization choice, particularly given that cultural values typically include additional fundamental dimensions beyond the individualism-collectivism spectrum.

12 In the first paragraph of the discussion, the author needs to summarize the basic results of this study.

While the manuscript currently has several issues that need addressing, I strongly encourage the authors to undertake further revisions, and I look forward to seeing these concerns adequately resolved in their next submission. Finally, I would like to emphasize that rest intolerance is indeed a highly significant issue worthy of in-depth investigation, particularly within the Chinese cultural context where it may present unique manifestations and implications that warrant special scholarly attention.
