# Supplementary material for: When winning costs your peace: How does vertical individualism Hijack relaxation capacity? Network analysis and mediation models
Source: PLoS One. 2025 Nov 6;20(11):e0335851. doi: 10.1371/journal.pone.0335851 (PMC12591470; doi:10.1371/journal.pone.0335851)
Supplement: Attachment — Submitted filename: [PONE-D-25-26911] respone_to_comments.docx [file pone.0335851.s008.docx]

**Dear Editor,**

Thank you very much for your comments. I have revised the manuscript according to the PLOS ONE formatting requirements and uploaded the updated version. In addition, the information on title, authors, and affiliations has been provided as a separate file ([PONE-D-25-26911] title, author, affiliations information). All text related to funding has been removed, and the contribution of the project sponsor, Haoyu Wang, has been specified in the Author Contributions section. We are in the process of submitting an application to the database website to make the data openly accessible. The DOI is <https://doi.org/10.57760/sciencedb.psych.00638>. The citation of Figure 1 has also been updated in the main text.

Below, please find our detailed responses to each of your comments. All revisions have been carefully highlighted in the file “Revised manuscript with track changes.” We sincerely appreciate your constructive guidance once again.

**To Editor:**

*We sincerely appreciate the valuable feedback provided by you and the reviewers for our manuscript PONE-D-25-26911, titled "When Winning Costs Your Peace: How Does Vertical Individualism Hijack Relaxation Capacity? Network Analysis and Mediation Models." We are deeply grateful for the time and effort you and the reviewers have dedicated to the review process, as well as for the insightful comments you have shared.*

*The suggestions were highly constructive and immensely helpful in refining our research and enhancing the quality of the manuscript. In response to all the comments, we have conducted a comprehensive and meticulous revision of the paper.*

*Below, we provide point-by-point responses to the editor's feedback, with all modifications clearly highlighted/marked in blue in the revised manuscript.*

**Comment 1:** Each of the four cultural dimensions needs some clear description in the Introduction, so readers can have a clearer understanding of each and more appreciation of the differences among them.

**Response:** Thank you for this valuable suggestion. We agree that providing clear definitions for the four cultural dimensions is crucial for readers to fully appreciate the theoretical foundation of our study. Accordingly, we have added a new paragraph in the **Introduction** section to explicitly define Horizontal Individualism (HI), Vertical Individualism (VI), Horizontal Collectivism (HC), and Vertical Collectivism (VC), based on the work of Triandis & Gelfand (1998).

These changes can be found on **Lines 81-91** of the revised manuscript.

**Comment 2:** I believe that this study is primarily exploratory in nature and the two stated hypotheses were not specific about the exact independent variable(s). As such, it’d be more appropriate to frame these statements as research questions rather than hypotheses. Then, for the rest of the manuscript, please make appropriate changes for all instances when a hypothesis is being mentioned.

**Response:** Thank you for this insightful recommendation. We agree entirely that framing our study with research questions is more appropriate given its exploratory nature. This change better reflects the goal of our study, which is to first identify the key cultural dimension related to rest intolerance and then to explore the underlying mechanism.

We have now reframed our hypotheses as research questions in the **Introduction 1.3** section. Furthermore, we have carefully revised the entire manuscript to ensure that all language related to "hypotheses" has been updated to align with the new "research question" framework.

The original hypotheses and the new research questions are as follows:

**Original Hypotheses:**

*H1: Specific dimensions of cultural values may significantly influence experiences of rest intolerance.*

*H2: Status anxiety may function as a mediating variable between one or more dimensions of cultural values and rest intolerance.*

**New Research Questions:**

***Q1:*** *Among the four dimensions of cultural values (HI, VI, HC, VC), which dimension is most strongly associated with rest intolerance in Chinese university students?*

***Q2:*** *Does status anxiety mediate the relationship between the key cultural value dimension identified in Q1 and rest intolerance?*

These changes can be found on **Lines 143-150, Figure 1, 243** of the revised manuscript).

**Comment 3:** Please provide sample items for each scale (or subscale), corresponding to the variables used in the analyses.

**Response:** Thank you for this suggestion to enhance the clarity of our methodology. We agree that providing sample items helps readers better understand the constructs we measured. We have revised the **2.2. Instruments** section to include a sample item for each scale and/or its dimensions.

For instance, in the descriptions for the Rest Intolerance Scale, the Individualism-Collectivism Scale, and the Status Anxiety Questionnaire, we have now added representative examples (e.g., for Rest Intolerance Scale, a sample item such as " When I am resting or having fun, I feel guilty.") to illustrate the content of the measures.

These changes can be found on **Lines 174-178, 184–187, and 196** of the revised manuscript.

**Comment 4:** For the cultural orientation scale, please report alpha for each of the four subscales.

**Response:** Thank you for pointing this out. We agree that reporting the individual Cronbach's alpha coefficients for each subscale is more precise and informative than providing a range. We have amended this in the **2.2.3. Individualism-Collectivism Scale** subsection. The manuscript now reports the specific reliability coefficient for each of the four dimensions: Horizontal Individualism (*α* = 0.641), Vertical Individualism (*α* = 0.684), Horizontal Collectivism (*α* = 0.752), and Vertical Collectivism (*α* = 0.698).

These changes can be found on **Lines 190-193** of the revised manuscript.

**Comment 5:** Descriptive statistics – For gender, reporting mean and SD do not seem appropriate (Figure 2a). Please report proportions. Moreover, for better understanding of your sample, please report descriptive statistics for other demographic variables (in addition to gender and age) that you have measured.

**Response:** Thank you again for this constructive feedback. We completely agree that frequencies and percentages are the appropriate way to describe the composition of our sample for categorical demographic variables. To address this, we have now added a detailed descriptive paragraph at the beginning of the **Results section (3.2)**, which reports the frequencies and percentages for gender, educational level, and academic major.

Regarding the table (now **Table 1** **Descriptive Statistics and Intercorrelations for Key Variables)**, its primary purpose is to provide a comprehensive overview of the intercorrelations among all variables as they were entered into our subsequent mediation analyses, where categorical variables were numerically coded. We have retained this table to ensure transparency regarding the data structure used in our models. We believe this dual approach—providing clear descriptive statistics in the text for sample composition and retaining the table for an analytical overview—best addresses your concern for clarity while maintaining the integrity of our full analytical report.

We also wish to sincerely apologize for an oversight in our original manuscript. The **2.2.1. Demographic variables** section previously mentioned that we collected data on "location of schooling" and "only child status." This was an error in the description, as this information was not collected in the final version of our survey. We have now removed this statement from the Methods section to accurately reflect the data that were actually collected and analyzed.

These changes can be found on **Lines169-171, 233-237, Table 1** of the revised manuscript.

**Comment 6:** Section 3.2 (Descriptive statistics and correlations): This section first reports regression results (Beta and p-value) for VI predicting SA and VI predicting RIS. Then, it describes a “correlation line test” with some positive and negative correlations. From the description and Figure 2b (“Pearson correlation diagram”), I assume these are raw Pearson correlations. If so, please provide a clear Pearson correlation table with r and p-values for all pairs (some correlations are very difficult to read). In the main text, you could then describe all significant correlation results with r and p values (reporting regression results will then become unnecessary in this subsection about descriptive statistics and correlations as they will be redundant), and direct readers to the full table. If there is any misunderstanding on my part, please clarify and still provide relevant statistics whenever a result is described in words.

**Response:** Thank you for this detailed and helpful guidance. Your understanding is entirely correct, and we acknowledge that the original presentation in this section was confusing and mixed different types of analyses. We have thoroughly revised this section according to your suggestions to improve its clarity and logical flow.

Specifically, we have made the following changes:

1. We have replaced the previous correlation diagram (Figure 2b) with a comprehensive correlation table **(now Table 1)**. This table clearly presents the Pearson's *r* coefficients for all pairs of variables, with significance levels (*p*-values) indicated by asterisks.
2. In the main text of **Section 3.2**, we have removed the preliminary regression results (β-values) to avoid redundancy and confusion.
3. The text now concisely describes the most important significant correlations from the table, reporting their specific *r* and *p*-values. We also direct the reader to Table 1 for the full correlation matrix.

These changes can be found on **Lines 245-250, Table 1** of the revised manuscript.

**Comment 7:** Section 3.3 (Network analysis result): Please provide relevant statistics whenever a result is described in words. Thus, in the sentence “…significant positive correlations between the "vertical individualism" dimension and both status anxiety and rest intolerance…”, it would be helpful to report the relevant statistics in the main text.

**Response:** Thank you for this important reminder to enhance the precision of our results reporting. We completely agree that results described in the text should be accompanied by their corresponding statistics.

We have revised Section 3.3 to include the specific edge weight values for the key positive correlations identified in our new 9-node network. For instance, we now report the specific weights for the connections between Vertical Individualism and Status Anxiety (edge weight = 0.30), as well as its primary links to the Rest Intolerance community. We have also reviewed the manuscript to ensure that other key findings are similarly supported by their relevant statistics in the text.

These changes can be found on **Lines 260-267, 272-274** of the revised manuscript.

**Comment 8:** Section 3.4 (Mediation effect analysis): Please also report the standardized coefficient (beta) for the indirect effect, in addition to the confidence interval. It mentions that the indirect effect accounts for “approximately 20% of the total effect”. It would be better to report the exact ratio of the indirect effect to the total effect without using “approximately”. For Figure 5a, it would be helpful to indicate in the caption what the number in brackets represents. You may also consider adding the statistics for the indirect effect in the figure to make it more complete. For the reverse mediation model, please report all relevant statistics, paralleling the results reported for the original mediation model. For Figure 5b, please make the same changes as Figure 5a.

**Response:** Thank you for this very detailed and constructive feedback on our mediation analysis reporting. We have carefully revised **Section 3.4** and the corresponding figure according to all of your suggestions to enhance their precision and completeness.

The specific changes are as follows:

1. **Reporting of Primary Model:** For the primary model (VI → SA → RIS), we have updated the text to include the standardized coefficient for the indirect effect (β = .10), the exact ratio of the indirect effect to the total effect (19.23%), and have added t-values for all path coefficients, as requested.
2. **New Exploratory Analyses:** Taking your suggestion for greater rigor to heart, we went a step further. Instead of simply reporting the reverse mediation model, we conducted four new exploratory mediation analyses using each of the four sub-dimensions of Rest Intolerance as outcome variables.
3. **New Comprehensive Figure:** Combined with your comments and those of the two reviewers. We have replaced the old (**Figure 5**) with a new, multi-panel **Figure 4 (a-e)** that presents the results of all five mediation models. Each subfigure now clearly displays the indirect effect value.
4. **Revised Figure Caption:** The caption for Figure 4 has been completely rewritten to explain the structure of the new figure, clarifying that the value on the path represents the direct effect (c') while the value in parentheses represents the total effect (c).

These changes can be found on **Lines 328-329, 343-344, Figure 4** of the revised manuscript.

**Comment 9:** Section 4.2 (Mediation model of vertical individualism, status anxiety, and rest intolerance): Repeating statistics that have been reported earlier is not necessary in the Discussion section.

**Response:** Thank you for this helpful advice on improving the structure of our discussion. We already delete this part and redesign the Discussion section.

**Comment 10:** As a partial mediation was found, speculations around what other mechanisms, in addition to status anxiety, might explain the relationship between VI and rest intolerance could be discussed in the Discussion section.

**Response:** Thank you for this insightful suggestion to deepen our discussion. This is an excellent point. The finding of partial mediation indeed suggests a more complex mechanism at play. We have now added a new paragraph to the end of **Section 4.2** speculating on other potential mediators, such as **performance-contingent self-worth** and the **perception of time as an opportunity cost.**

These changes can be found on **Lines 392-399** of the revised manuscript.

We sincerely hope that the revised version meets the publication standards of *PLOS ONE*.

**To Reviewer #1:**

*First and foremost, please allow us to express our most sincere and heartfelt gratitude. Your review of our manuscript went far beyond a standard critique; for us, it felt like a profound and generous academic mentorship. Each of your comments was incredibly insightful, pinpointing the precise weaknesses in our study and illuminating the path for its improvement.*

*We are especially grateful for your guidance on the network analysis. Your suggestions did more than just help us correct our methods; they inspired us to go back, relearn, and conduct a much deeper and more meaningful analysis. The revision process, guided by your feedback, has been an invaluable learning experience for which we are truly thankful. We hold the deepest respect for the intellectual labor and scholarly insight you have so generously shared with us.*

*Below, we provide point-by-point responses to the reviewers' feedback, with all modifications clearly highlighted/marked in red in the revised manuscript.*

**Comment 1:** In lines 67-71, the author states that "This conflict may significantly intensify the experience of rest intolerance." However, from my perspective, individual achievement itself can be viewed as a means of contributing to the collective... The conflict mentioned by the author doesn't appear to be a definitive causal factor.

**Response:** Thank you for this very insightful point. We completely agree with your assessment. The relationship between individualism and collectivism in the contemporary Chinese context is indeed complex and not one of simple opposition. Your point that "individual achievement itself can be viewed as a means of contributing to the collective" is excellent and has prompted us to refine our argument.

Following your suggestion, we have revised this section to adopt a more nuanced perspective:

1. We have replaced the strong and potentially misleading term **"conflict"** with phrases like "**psychological tension**" or "**complex interplay**" to better capture the dynamic.
2. We have rewritten the paragraph to first acknowledge that individual achievement can serve collective goals (e.g., honoring one's family). We then clarify that the tension arises more specifically from the perceived dichotomy between **personal leisure** (which can be viewed as purely self-serving) and the relentless pressure for **achievement** (which serves both individual and collective ends).
3. We also agree that the causes of rest intolerance are multifaceted, and we have **softened** our causal language to present this cultural tension as one significant contributing factor among others, rather than the sole definitive cause.

These changes can be found on **Lines 71-78** of the revised manuscript.

**Comment 2:** The statement in line 87, "The relationship between cultural values and rest intolerance may be influenced by additional factors," clearly suggests a moderating effect rather than mediation as the author claims... The author appears to have misapplied these fundamental statistical concepts in their interpretation.

**Response:** Thank you for your sharp observation and for pointing out this critical lack of clarity. We sincerely apologize for this imprecise wording. You are entirely correct that the phrase "the relationship... may be influenced by" describes a moderating effect. This was a poor choice of words on our part, which inadvertently misrepresented our research question.

Our theoretical framework and subsequent analyses were indeed designed to test the mediating role of status anxiety—that is, to examine it as an intervening mechanism through which cultural values exert their influence on rest intolerance (VI → SA → RIS).

To correct this and remove any ambiguity, we have completely rewritten the introductory sentence of this section to accurately describe a mediation pathway.

We are grateful for your diligence in identifying this issue, as the correction greatly improves the conceptual precision of our manuscript.

These changes can be found on **Lines 105-110** of the revised manuscript.

**Comment 3:** In lines 157-158, it appears the author intended to refer to "horizontal individualism, vertical individualism, horizontal collectivism, and vertical collectivism" (rather than the terms currently presented in the text).

**Response:** Thank you for your careful reading and for catching this error. You are absolutely correct. This was a typographical error on our part. We have now corrected this sentence in **Section 2.2.3** to accurately list the four dimensions as "horizontal individualism, vertical individualism, horizontal collectivism, and vertical collectivism."

These changes can be found on **Lines 185-188** of the revised manuscript.

**Comment 4:** Regarding lines 214-215, please maintain consistency in the terminology for the dependent variable throughout the manuscript. While "rest intolerance" may also be referred to as "resting shame" in the Chinese academic context, the English version should use a single, standardized term (preferably "rest intolerance") for reader comprehension. This inconsistency also appears in lines 217 and 219. We recommend a thorough proofread of the entire manuscript to ensure terminological consistency for this key construct.

**Response:** Thank you very much for your meticulous attention to detail and for pointing out this important inconsistency. You are correct that the term was used inconsistently in the lines you indicated. During our revisions for the Academic Editor, we had already standardized this term in several places and deleted some similar expressions. We are very grateful that you caught the remaining instances.

Prompted by your comment, we conducted a final, thorough proofread of the entire manuscript. We have now corrected the lines you pointed out, and a final check revealed one last oversight on line 239, which has also been amended. All instances have now been standardized to the official term "rest intolerance" to ensure complete consistency throughout the paper.

These changes can be found on **Line 246** of the revised manuscript.

**Comments 5 & 9:** Please revise the legend for Figure 3. The current inclusion of "Rest Intolerance" in the figure legend appears functionally unnecessary as it does not contribute meaningful explanatory value to the graphical presentation. //Why not control for covariates in network analysis?

**Response:** Thank you for your suggestions regarding the visual presentation and the analytical rigor of our network model. We have addressed both points in our revision.

Regarding the legend for **Figure 3 (now Figure 2 & 3) (Comment 5)**, we have proactively revised the figure. We removed the previous grouping and assigned a unique color to each node. The legend now serves to clearly and independently identify the full variable name for each uniquely colored node. We hope this revised presentation is clearer and more effective.

Regarding the inclusion of covariates **(Comment 9),** this is an excellent and critical point. In our initial exploratory analysis, we did not control for covariates. However, we completely agree that controlling for potential confounders makes the network model more robust. Following your invaluable suggestion, we have rerun our network analysis, this time controlling for the demographic variables that showed significant correlations with our key variables—specifically, **gender, edu, and age** were included as covariates. We have updated **the Section 3.2** and **the caption for Figure 2** to reflect this significant methodological improvement. We are very grateful for this suggestion, as it has substantially strengthened our study.

These changes can be found on **Lines 251-253, 257-258, 324** of the revised manuscript.

**Comment 6:** In line 217, the author states "a positive correlation between resting shame and status anxiety," implying a positive association between rest intolerance and status anxiety. However, in line 177, the author specifies that "Positive and negative associations are represented by blue and red edges, respectively." This creates a contradiction, as the figure depicts rest intolerance and status anxiety connected by a red edge (indicating a negative correlation), which is inconsistent with the textual description.

**Response:** Thank you for your exceptional attention to detail in catching this critical inconsistency. We sincerely apologize for this error. The error was in the network visualization, where the edge was incorrectly rendered as red. We have now corrected **Figure 2**, and the edge connecting rest intolerance and status anxiety is now correctly colored **blue** to represent a positive association, consistent with both the text and our color key.

These changes can be found on **Line 309** of the revised manuscript.

**Comment 7:** The phrasing in lines 218-219 – "a negative correlation between resting shame and status anxiety and horizontal collectivism" – is structurally ambiguous and may lead to misinterpretation. It remains unclear whether: 1) resting shame correlates negatively with both status anxiety and horizontal collectivism, or 2) resting shame and status anxiety collectively correlate negatively with horizontal collectivism.

**Response:** Thank you for pointing out this ambiguity. We agree that the original sentence was poorly phrased and confusing. Our intention was to state that horizontal collectivism was negatively correlated with both rest intolerance and status anxiety separately. We have rewritten this sentence in **Section 3.2** to be clear and unambiguous.

These changes can be found on **Lines 246-251** of the revised manuscript.

**Comment 8:** The centrality analysis in Section 3.3 attempts to identify the most central node in the network, but unfortunately fails to achieve its intended purpose. Rather than examining global network centrality - which is inherently influenced by node selection bias - the analysis should focus on bridge centrality metrics between the rest intolerance and individualism-collectivism subnetworks, as these would provide more meaningful insights into the core research questions. The authors must further justify their node selection strategy, particularly explaining why rest intolerance was treated as a unitary construct rather than being operationalized through its constituent dimensions. A critical remaining question is how the network structure and centrality findings might differ if the multidimensional components of rest intolerance were modeled as separate nodes in the analysis.

**Response:** Thank you for this exceptionally insightful and constructive critique of our network analysis. We completely agree that our initial approach could be significantly improved, and your suggestions have prompted us to conduct a new, more granular, and far more informative analysis. Instead of merely justifying our previous method, we have fully embraced your recommendation to explore the multidimensional nature of rest intolerance.

1. **New Analysis with Sub-dimensions:** We have **completely re-run our network analysis** using the four sub-dimensions of the Rest Intolerance Scale (RIS) as individual nodes (Negative Feelings, Social Comparison, Obsessive Thinking, Cognitive Bias). The new network now consists of nine nodes in total, allowing for a much more detailed examination of the relationships.
2. **Bridge Centrality Analysis:** As you correctly pointed out, bridge centrality is the more appropriate metric. We performed a bridge centrality analysis on this new 9-node network, defining three communities: "Individualism-Collectivism" (4 nodes), "Status Anxiety" (1 node), and "Rest Intolerance" (the 4 RIS sub-dimensions).
3. **New, Richer Findings:** This deeper analysis has yielded much richer insights. As reported in the revised **Section 3.3**, our new results show that **Vertical Individualism (VI) and Status Anxiety (SA) act as the most critical bridge nodes** connecting the cultural values community to the rest intolerance community. The revised manuscript now includes a detailed report on these bridge centrality findings, stability tests for the new network (including CS-coefficient for bridge strength = 0.60), and a new figure (Figure 3) that visually highlights these core bridge nodes.

We have thoroughly updated our **Methods (Section 2.3)**, **Results (Section 3.3)**, and **Discussion** to reflect this new, more sophisticated analysis. We are sincerely grateful for your suggestions.

These changes can be found on **Lines 202–224, 255–306, Figure 2 & 3, Appendix Figure 1-4** of the revised manuscript.

**Comment** **10:** While the use of cross-sectional mediation in this exploratory study may be marginally acceptable, the proposed mediation pathways among individualism-collectivism, status anxiety, and rest intolerance hold limited substantive value. Given that cross-sectional mediation can produce statistically significant effects based solely on correlations—without establishing temporal precedence or causal direction—the reported findings require stronger theoretical justification.

**Response:** Thank you for this critical and important point regarding the limitations of cross-sectional mediation. We completely agree that our design does not allow for causal claims. Our intention for the mediation analysis is not to establish causality, but rather to serve as a plausibility test for a specific, theoretically-driven pathway that was suggested by our primary network analysis.

We have revised the manuscript to make this framing explicit. We now argue that the network analysis provides a broad, structural map of the relationships, while the mediation analysis offers a focused test on the most plausible directional pathway (VI → SA → RIS), which is justified by both theory (i.e., stable cultural values preceding transient psychological states) and our bridge centrality findings. We have also added a more detailed acknowledgment of this limitation in the Discussion section. We believe this complementary methodological approach provides a richer, albeit exploratory, understanding of the phenomenon.

These changes can be found on **Lines 317-321, 382-386**of the revised manuscript.

**Comment** **11:** As a non-specialist in cultural value research, I find it unclear whether individualism-collectivism can adequately serve as a representative measure of cultural values, or how precisely these two constructs differ and relate to each other conceptually. This distinction is particularly important as the manuscript appears to use these terms interchangeably without proper clarification, potentially conflating the specific dimension of individualism-collectivism (which focuses on self-group relations) with the broader, multidimensional construct of cultural values that encompasses various societal orientations beyond just this single aspect. The authors should explicitly address this conceptual overlap and justify their operationalization choice, particularly given that cultural values typically include additional fundamental dimensions beyond the individualism-collectivism spectrum.

**Response:** Thank you for this crucial observation. We agree that our initial draft used the broad term 'cultural values' and the specific framework of 'individualism-collectivism' interchangeably, which could lead to conceptual ambiguity. To address this, we have systematically revised the introduction to clarify our approach. We now explicitly frame individualism-collectivism and its vertical-horizontal dimensions as the specific, measurable cultural value orientations under investigation in this study. The terminology throughout the introduction has been updated to consistently reflect that we are examining these specific dimensions as a way to understand the broader influence of cultural values.

These changes can be found on **Lines 61-63, 67-69, 103, 119** of the revised manuscript.

**Comment** **12:** In the first paragraph of the discussion, the author needs to summarize the basic results of this study.

**Response:** Thank you for this essential suggestion. We agree that beginning the discussion with a concise summary of the main findings is crucial for clarity and impact. We have rewritten the opening paragraph of the **Discussion section (Section 4)** to provide a clear overview of the key results from both our network and mediation analyses.

These changes can be found on **Lines 347-355** of the revised manuscript.

**To Reviewer #2:**

**Comment:** Rest intolerance is an interesting topic, but I found the writing style to be too dense. Perhaps the authors could try to simplify what they are trying to say. A more elaborate definition of vertical collectivism would be useful for example. In it’s present form it is hard to read.

**Response:** Thank you for this valuable feedback regarding the manuscript's readability and logical flow. We sincerely appreciate your constructive suggestions. We agree that the previous version of the manuscript was dense and could be clearer.

Following your advice, we have undertaken a significant revision to restructure the paper's narrative and clarify its core arguments. The analytical logic of the paper has been reorganized to follow a more intuitive, step-by-step progression:

1. We begin with a broad **network analysis** to explore the overall structure of relationships among all variables.
2. Next, we employ **bridge centrality analysis** to identify the most critical "bridge variables" that connect the different psychological communities.
3. Finally, based on the key bridge nodes identified, we conduct a **mediation analysis** to formally test a specific, theoretically plausible pathway.

We believe this new structure makes the paper's central argument much easier to follow. Additionally, as you suggested, we have added a more elaborate definition of **vertical collectivism**, along with the other three cultural dimensions, in the Introduction section to ensure readers can better appreciate the distinctions. We hope these comprehensive revisions have made the manuscript significantly clearer and more accessible.

These changes can be found on **Lines 81-91, 323-338** of the revised manuscript.

----END----
